# Can Fraction of Inspired Oxygen Predict Extubation Failure in Preterm Infants?

**DOI:** 10.3390/children9010030

**Published:** 2022-01-01

**Authors:** Eugenio Spaggiari, Maria Amato, Ornella Angela Ricca, Luigi Corradini Zini, Ilaria Bianchedi, Licia Lugli, Alessandra Boncompagni, Isotta Guidotti, Cecilia Rossi, Luca Bedetti, Lorenzo Iughetti, Alberto Berardi

**Affiliations:** 1 Neonatal Intensive Care Unit, Women’s and Children’s Health Department, Policlinico University Hospital of Modena, Via del Pozzo 71, 41124 Modena, Italy; spaggiari.eugenio@aou.mo.it (E.S.); lugli.licia@aou.mo.it (L.L.); boncompagni.alessand@aou.mo.it (A.B.); guidotti.isotta@aou.mo.it (I.G.); rossi.cecilia@aou.mo.it (C.R.); luca.bedetti87@gmail.com (L.B.); 2 Pediatric Post-Graduate School, Women’s and Children’s Health Department, University Hospital of Modena and Reggio Emilia, 41124 Modena, Italy; mariaamato11@gmail.com (M.A.); riccaornellaangela@gmail.com (O.A.R.); bianchedi.ilaria@gmail.com (I.B.); lorenzo.iughetti@unimore.it (L.I.); 3 Medical School, University of Modena and Reggio Emilia, 41121 Modena, Italy; l.zini@libero.it; 4 PhD Program in Clinical and Experimental Medicine, University of Modena and Reggio Emilia, 41121 Modena, Italy; 5 Pediatrics, Women’s and Children’s Health Department, University of Modena, 41121 Modena, Italy

**Keywords:** extubation failure, preterm infant, fraction of inspired oxygen, mechanical ventilation

## Abstract

Background: Prolonged mechanical ventilation in preterm infants may cause complications. We aimed to analyze the variables affecting extubation outcomes in preterm infants at high risk of extubation failure. Methods: This was a single-center, observational, retrospective study. Extubation failure was defined as survival with the need for reintubation within 72 h. Successfully extubated neonates (group 1) were compared to those with failed extubation (group 2). Multivariate logistic regression analysis evaluated factors that predicted extubation outcomes. Results: Eighty infants with a birth weight under 1000 g and/or gestational age (GA) under 28 weeks were included. Extubation failure occurred in 29 (36.2%) and success in 51 (63.8%) neonates. Most failures (75.9%) occurred within 24 h. Pre-extubation inspired oxygen fraction (FiO_2_) of 27% had a sensitivity of 58.6% and specificity of 64.7% for extubation failure. Post-extubation FiO_2_ of 32% had a sensitivity of 65.5% and specificity of 62.8% for failure. Prolonged membrane rupture (PROM) and high GA were associated with extubation success in multivariate logistic regression analysis. Conclusions: High GA and PROM were associated with extubation success. Pre- and post-extubation FiO_2_ values were not significantly predictive of extubation failure. Further studies should evaluate if overall assessment, including ventilatory parameters and clinical factors, can predict extubation success in neonates.

## 1. Introduction

Mechanical ventilation (MV) increases survival in preterm infants but may lead to complications when prolonged unnecessarily. Bronchopulmonary dysplasia, neurodevelopmental and growth impairments, as well as increased risks of infections or mortality, may occur after prolonged MV [1,2,3]. Furthermore, prolonged endotracheal intubation is associated with the risk of upper airway damage (such as laryngeal edema, subglottic stenosis, and tracheomalacia) [4,5,6]. Thus, extubation should be performed as soon as possible if the baby is “ready for extubation”. However, extubation failure may lead to increased ventilatory support, bradycardia, oxygen desaturations, large fluctuations in blood and intracranial pressure, lung derecruitment, and upper airway injury during reintubation attempts [7]. Currently, there are no standardized recommendations for the safe extubation of preterm neonates. Indications to extubate neonates are heterogeneous and may vary across centers [8]. The timing of extubation often depends on clinical judgment, after taking into account the birth weight (BW), gestational age (GA), ventilator parameters, blood gas analysis, chest X-rays, and breathing tests. However, these clinical variables were singularly shown to have a low predictive value for extubation failure in previous studies [9,10,11,12,13,14]. Furthermore, most of these studies addressed neonates with a wide range of GA and BW, although the risk of extubation failure is the highest among preterm neonates with an extremely low GA and BW. The aim of this study is to analyze the predictive factors for extubation failure in a cohort of extremely preterm and/or extremely low birth weight infants.

## 2. Materials and Methods

This was a retrospective, observational, single-center study carried out at the University Hospital of Modena, Italy. Preterm neonates with a birth weight under 1000 g and/or gestational age under 28 weeks who were admitted to the neonatal intensive care unit (NICU) from 1 December 2010 to 31 December 2019 were enrolled. Data regarding the first extubation attempt were evaluated. Inclusion criteria were intubation in the first 24 h of life and at least 12 h of conventional or high frequency oscillatory mechanical ventilation. Exclusion criteria were intubation after the first 24 h of life, unplanned extubation, outborn neonates, or neonates with major congenital malformations. As per practice at our center, surfactant was administered if the newborn required a fraction of inspired oxygen (FiO_2_) over 30% or had signs of severe respiratory distress. If the newborn was already intubated, surfactant was administered by endotracheal tube and ventilation was continued. Neonates on non-invasive respiratory support received surfactant by INtubation-SURfactant-Extubation procedure. Newborns were extubated when they were deemed “clinically ready for extubation”. This evaluation was usually based on the stability of vital signs, good spontaneous respiratory activity, maximum peak inspiratory pressure under 16 cm H_2_O on conventional ventilation with respiratory support rate under 30 bpm, or mean airway pressure under 10 cm H_2_O on high-frequency oscillatory ventilation. If volume guarantee was used, it was set to 4–6 mL/kg on conventional ventilation and to 1.5–2.5 mL/kg on high-frequency oscillatory ventilation. Intravenous caffeine (20 mg/kg within 60 min before extubation) was administered to all infants before extubation. 

After extubation, newborns usually underwent non-invasive respiratory support with nasal continuous positive airway pressure (CPAP) (from 5 to 7, up to 9 cm H_2_O) or nasal bilevel CPAP (usually high/low pressure from 9/6 cm H_2_O up to 12/8 cm H_2_O, T-high 1 s, rate 30). Criteria for reintubation were (i) recurrent apnea (4 or more episodes in one hour or >2 episodes of apnea requiring bag and mask ventilation), (ii) respiratory acidosis (pH < 7.2 with pCO_2_ > 65 mmHg), or (iii) increased work of breathing associated with an increased need for oxygen support (defined as level of oxygen) to achieve a saturation target of 90–95%. Apnea was defined as cessation of breathing associated with an immediate drop in oxygen saturation and bradycardia.

Data were extracted from the electronic chart “Meta Vision Suite—iMDsoft^®^ version 5.46.44”. For each case, we collected patient and maternal demographics, as well as the neonatal characteristics at the time of extubation. 

Pre-extubation FiO_2_ refers to the mean FiO_2_ reported during the 2 h prior to the extubation attempt; post-extubation FiO_2_ refers to the maximum FiO_2_ supplied during the first 24 h after the extubation attempt. According to the main outcome, neonates were divided into two groups: group 1, extubation success and group 2, extubation failure. Extubation that required reintubation within the following 72 h was considered a “failure”. The study was approved by the institutional ethics review board (No. 330/2017).

### Statistical Analyses

Statistical analyses were performed by using MedCalc, version 9.3 (MedCalc Software, Ostend, Belgium). Continuous variables were expressed as means ± standard deviations or medians and ranges. Categorical data were expressed as percentages. Student’s *t*-test and Mann–Whitney rank sum test were used to compare the continuous variables, while the χ^2^ test or Fisher’s exact test was used to compare the categorical variables between the two groups. The area under the receiver operating characteristic curve (AUC) was also calculated. A *p* value < 0.05 was considered as the threshold for statistical significance, while a *p* value > 0.05 and <0.1 was the indicator of a trend. 

Prenatal and postnatal clinical characteristics were evaluated as possible risk factors for extubation failure. In order to calculate the median increase of FiO_2_ between pre- and post-extubation, we created a variable (DeltaFiO_2_). This is the difference between FiO_2_ post-extubation and FiO_2_ pre-extubation, divided for FiO_2_ pre-extubation, finally multiplied by 100. All maternal and neonatal characteristics were included in univariate logistic regression analysis. Subsequently, a multivariate logistic regression model was built on the basis of stepwise selection, with entry criteria = 0.05 and stay criteria = 0.1 (variables included were: gestational age, pre-extubation FiO_2_, post-extubation FiO_2_, days on mechanical ventilation, and prolonged membrane rupture).

## 3. Results

**Figure 1:**
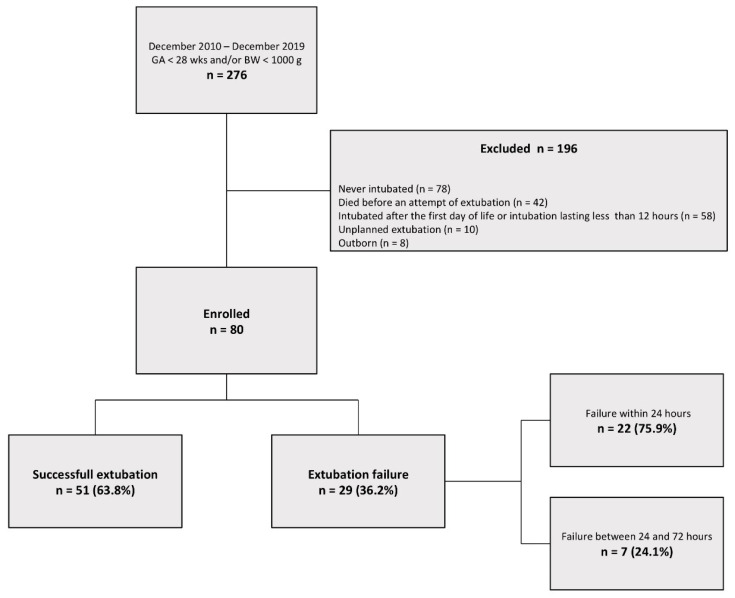
Flow chart of study population selection.

A total of 276 neonates with a gestational age under 28 weeks and/or birth weight under 1000 g were admitted to the NICU during the study period. The mean gestational age was 25.92 weeks (standard deviation [SD] 1.45). The average weight was 809.98 g (SD 171.67). The flowchart of patients’ selection is shown in Figure 1.

Upon 80 infants enrolled in the study, 68 (85%) were intubated in the delivery room according to international newborn life support guidelines [15,16], while 12 (15%) were intubated within the first 24 h of life due to apneas or increased work of breathing. All infants received surfactant within the first 24 h of life. Thirty-one out of eighty neonates received one dose of surfactant in the delivery room immediately after intubation.

Extubation failed in 29/80 (36.2%) neonates and was successful in 51/80 (63.8%) neonates. The reasons for extubation failure were recurrent apneas (31%) or increased work of breathing associated with an increased need for oxygen support (69%). Most failures (75.9%) occurred within 24 h. A comparison of the maternal and neonatal characteristics between infants in group 1 and group 2 is shown in Table 1. 

On comparing the two groups, there were significant differences in the frequency of prolonged membrane rupture (PROM), median pre-extubation FiO_2_, and median days of mechanical ventilation. Median post-extubation FiO_2_ was higher in infants with extubation failure (*p* 0.05). Additionally, extubation failure increased by 5% for each additional day of mechanical ventilation.

The prediction of FiO_2_ for extubation failure was investigated, and all FiO_2_ values were analyzed. The best pre-extubation FiO_2_ cut-off value was 27%, with a sensitivity of 58.6% and a specificity of 64.7%; this value correctly predicted 62.5% of extubation failures, and the AUC was 0.68. The best post-extubation FiO_2_ cut-off value was 32%, with a sensitivity of 65.5% and a specificity of 62.8%; this value correctly predicted 63.8% of extubation failures, and the AUC was 0.63. DeltaFiO2 was assessed: the median percentage increase in FiO_2_ was +20.0 (interquartile range (IQR) 4.8–42.9) when an extubation was successful, and +12.9 (IQR 0–38.5) when an extubation failed (*p* = 0.5).

In multivariate logistic regression analysis, only the absence of PROM and low GA were significantly associated with extubation success (AUC 0.81, *p* < 0.01). The risk of extubation failure decreased by 27% for each additional week of gestational age (Table 2).

## 4. Discussion

Assessing the parameters predictive of successful extubation is pivotal for improving the outcomes of neonates with a low GA and BW since prolonged mechanical ventilation is associated with a high risk of complications in this group [17,18].

Unlike most previous studies which evaluated infants with GA and BW ranging from 25 to 33 weeks and from 690 g to 1900 g, respectively [9,10,11,12], our study evaluated a very selected population of neonates with an extremely low BW and GA who have the highest risk of extubation failure. The successful extubation rate was 63.8%, which is consistent with those reported by previous studies, ranging from 50% to 73% [9,10,11,12]. However, it is difficult to compare studies that differ in terms of the analyzed population and definition of successful extubation. We used the cut-off value of 72 h for defining extubation failure as suggested in a recent review [18]. Indeed, in our opinion, 72 h is a sufficient period to establish if the newborn is able to breathe autonomously. Beyond this time frame, additional factors affecting breathing may arise in the first days of life (that is, infections, excessive changes in body weight, hemodynamic changes due to patent ductus arteriosus). 

We found that PROM was strongly associated with successful extubation. This finding is consistent with those of previous studies [9,19], although the investigators do not mention the possible causes of this association. Moreover, PROM is associated with histological chorioamnionitis in 40–70% of patients at lower gestational ages [19,20]. Chorioamnionitis can both injure and mature the fetal lung; some studies argue that chorioamnionitis may result in early lung maturation by decreasing the severity of respiratory distress syndrome thanks to increased surfactant production and decreased pro-inflammatory mediators in the fetal airways [21]. However, unexpectedly, we could not confirm an association between chorioamnionitis and successful extubation. Unknown factors triggered by PROM may affect lung maturation independently from chorioamnionitis, and this finding deserves additional investigation.

Previous studies [9,22] have reported an association between a low GA and BW and extubation failure. In our study, no association was found with BW, but a low GA was significantly associated with extubation failure in multivariate analysis. Although the cause for this discrepancy is unclear, GA likely reflects the degree of development of the infant’s lung and breathing pattern better than BW. Our findings are consistent with those of a recent study that examined successful extubation in neonates with an extremely low BW and GA undergoing high-frequency oscillatory ventilation [23].

In our study, pre- and post-extubation FiO_2_ values were higher in infants with extubation failure, although only pre-extubation FiO_2_ was significantly different between the two groups. However, it was not significantly associated with extubation failure in multivariate logistic regression analysis. These findings are consistent with those in existing literature [9,10,22]. A previous report [24], which focused on extremely low birth weight (ELBW) neonates, did not find a significant association between the FiO_2_ value and extubation failure, although median pre- and post-extubation FiO_2_ values were higher in infants with extubation failure.

There is wide variability in the FiO_2_ cut-off values used to predict extubation failure, although a median FiO_2_ cut-off value of 35% was considered an extubation criterion in a recent review [22]. We could not identify any pre- or post-extubation FiO_2_ cut-off values that were highly predictive of extubation failure. FiO_2_ values increased from pre- to post-extubation in both the failure and successful extubation groups. The amount of increase in FiO_2_ values was similar in both groups. These findings suggest that FiO_2_ alone is not sufficient for the decision-making process of extubation. Additional factors (that is, lung derecruitment, high airway resistance, recurrent apnea, respiratory muscle weakness, insufficient respiratory effort, hemodynamic instability) may affect the rate of extubation failure [11]. Identifying whether an infant is “ready for extubation” requires an overall assessment of the infant’s clinical condition rather than an evaluation of individual parameters. For example, spontaneous breathing test, post-extubation respiratory severity score, extubation success calculator, and lung ultrasound could also be used [25,26,27,28,29]. 

This study has a number of limitations. Firstly, this was an observational, retrospective study, and we were unable to collect detailed data on all respiratory and clinical parameters at the time of extubation (such as mean airway pressure, peak inspiratory pressure, oxygen index, pH, and pCO_2_). Furthermore, information about the timing of prenatal steroids was sometimes missing, which did not allow us to assess whether steroids had been given many weeks prior to delivery, thus decreasing the effectiveness [30,31]. Further factors (such as patent ductus arteriosus treatment) that were not investigated in this study could have affected extubation failure. Data collection in this study concern a 9-year period, and substantial changes in the management of preterm neonates may have occurred during this period, affecting the extubation outcomes. Finally, the sample size in this study was relatively small, potentially affecting the statistical significance of some results. 

## 5. Conclusions

This study investigated a selected population of infants at high risk of extubation failure. PROM and GA were found to be the significant predictive factors for successful extubation. Although higher pre-extubation FiO_2_ values were associated with failure, single FiO_2_ cut-off values demonstrated low sensitivity and specificity. Further studies are needed to evaluate if overall clinical assessment rather than single factors is more useful to guide extubation in neonates.

## Figures and Tables

**Table 1:** Maternal demographics and neonatal characteristics.

	Success (Group 1) n = 51	Missing Cases, n	Failure (Group 2) n = 29	Missing Cases, n	Total ^§^	*p* ^§^
Full course of prenatal steroids, n (%)	29 (56.86)	17	20 (68.96)	6	49 (85.96)	0.48
Prolonged membrane rupture > 18 h, n (%)	21 (41.17)	-	2 (6.89)	-	23 (28.75)	<0.01
Histological chorioamnionitis or funisitis, n (%)	25 (49.01)	-	11 (37.93)	-	36 (45.00)	0.34
Male sex, n (%)	31 (60.78)	-	20 (68.96)	-	51 (63.75)	0.46
Small for gestational age, n (%)	3 (5.88)	-	2 (6.89)	-	5 (6.25)	0.86
Median birth weight, g (IQR)	825 (700–937)	-	754 (635–940)	-	807 (670–938)	0.15
Median gestational age, wks (IQR)	26 (25–27)	-	26 (25–27)	-	26 (25–27)	0.09
Median 5 min apgar score (IQR)	7 (5–8)	-	7 (6–8)	-	7 (5.5–8)	0.27
Patent ductus arteriosus, n (%)	37 (72.54)	6	23 (79.31)	3	60 (84.50)	0.77
Median days of life at first extubation (IQR)	2 (1–6)	-	2 (1–6)	-	2 (1–6)	0.90
Postnatal pre-extubation steroids, n (%)	4 (7.84)	-	5 (17.24)	-	9 (11.25)	0.21
Median pre-extubation FiO_2_ (IQR)	25 (22–28)	-	27 (25–31)	-	26 (22–28)	0.01
Median post-extubation FiO_2_ (IQR)	30 (27–35)	-	35 (30–39)	-	28 (26.7–35.0)	0.05
DeltaFiO_2_ (IQR)	20 (4.8–42.9)	-	12.9 (0–38.5)	-	20 (0–40)	0.50
O2 at 36 weeks of gestational age, n (%)	11 (21.56)	8	7 (24.13)	5	18 (26.86)	0.75
ROP at discharge from hospital, n (%)	6 (11.76)	7	3 (10.34)	5	9 (13.23)	0.90
Median days on mechanical ventilation ^¶^ (IQR)	6 (2–10)	-	12 (8–19)	-	8 (4–13)	<0.01
In-hospital mortality, n (%)	9 (17.64)	-	5 (17.24)	-	14 (17.50)	0.96

FiO_2_—fraction of inspired oxygen; g—grams; IQR—interquartile range; ROP—retinopathy of prematurity; wks—weeks. ^§^ Total and significance calculated excluding missing cases. ^¶^ Median days on mechanical ventilation are referred to the entire duration of the hospitalization.

**Table 2:** Multivariate logistic regression analysis of significant variables.

	Odds Ratio	95% Confidence Intervals	*p*
Median gestational age	0.67	0.47–0.97	0.03
Prolonged membrane rupture >18 h	0.06	0.01–0.39	<0.01
Median pre-extubation FiO_2_	1.09	1.00–1.20	0.06

FiO_2_—fraction of inspired oxygen. Odds ratios are related to extubation failure. Odds ratio are related to extubation failure.

## Data Availability

Data are available upon request to the corresponding author.
